# Clinico-pathological characteristics and outcomes of patients with biopsy-proven hypertensive nephrosclerosis: a retrospective cohort study

**DOI:** 10.1186/s12882-016-0254-2

**Published:** 2016-04-11

**Authors:** Shaoshan Liang, Weibo Le, Dandan Liang, Hao Chen, Feng Xu, Huiping Chen, Zhihong Liu, Caihong Zeng

**Affiliations:** National Clinical Research Center of Kidney Diseases, Jinling Hospital, Nanjing University School of Medicine, East 305 Zhongshan Road, Nanjing, Jiangsu 210002 China

**Keywords:** Hypertension, Benign nephrosclerosis, Malignant nephrosclerosis, Risk factors, Renal survival

## Abstract

**Background:**

This study aimed to investigate renal outcomes and their predictors in biopsy-proven hypertensive nephrosclerosis (HN) patients and to compare clinico-pathological characteristics and prognoses between benign nephrosclerosis (BN) and malignant nephrosclerosis (MN) patients.

**Methods:**

Data for biopsy-proven HN patients were retrospectively analyzed. Renal survival rates and relationships between clinico-pathological characteristics and outcomes were assessed.

**Results:**

A total of 194 patients were enrolled; the mean age at biopsy was 43.8 years, and male gender predominated (82.5 %). The median duration of hypertension was 5.0 years, and the mean systolic and diastolic blood pressures were 195 ± 37 and 126 ± 26 mmHg, respectively. The median serum creatinine (Scr) level, estimated glomerular filtration rate (eGFR), and proteinuria level were 1.61 mg/dl, 49.6 ml/min/1.73 m^2^, and 0.80 g/24 h, respectively. BN and MN were found by renal biopsy in 55.2 % and 44.8 % of patients, respectively. At biopsy, MN patients were younger, and had higher median Scr and proteinuria levels, higher incidences of anemia, hypertensive heart disease and hypertensive retinopathy, and worse renal outcomes than BN patients. During a median follow-up period of 3.0 years, 36 patients (18.6 %) reached end-stage renal disease (ESRD), and the 5- and 10-year cumulative renal survival rates for HN patients were 84.5 % and 48.9 %, respectively. A decreased baseline eGFR, an increased baseline proteinuria level, anemia, increased percentage of global glomerulosclerosis and tubular atrophy and interstitial fibrosis (TAIF) were independent predictors of future ESRD.

**Conclusions:**

The clinico-pathological characteristics and prognoses were significantly different between the MN and BN patients. The renal outcomes of HN patients were independently associated with the baseline eGFR and proteinuria level, anemia, percentage of global glomerulosclerosis and TAIF.

**Electronic supplementary material:**

The online version of this article (doi:10.1186/s12882-016-0254-2) contains supplementary material, which is available to authorized users.

## Background

Hypertension is a worldwide public health challenge due to its high prevalence, occurring in up to 26 % of the adult population [1], and the concomitant risks of cardiovascular, cerebrovascular and kidney disease. Hypertensive nephrosclerosis (HN) is a common risk factor for end-stage renal disease (ESRD) in developed countries, accounting for 3.3-23.4 % of ESRD patients in Europe (according to the ERA-EDTA Registry Annual Report, 2011) and 30.5 % of ESRD patients in the US [2]. The incidence of ESRD is 2.7 per 100,000 person-years in the Chinese population [3]; thus, it is a great burden in this country because of the large population.

Usually, a diagnosis of HN is assigned based on clinical manifestations. Most studies of HN have focused on the clinical predictors of renal disease progression, including race [4, 5], blood pressure [6–9], renal dysfunction [elevated serum creatinine (Scr) level or decreased estimated glomerular filtration rate (eGFR)] [6, 10–13], proteinuria [10, 11], and concomitant cardiovascular disease [11, 14, 15]. Hypertension is divided into benign hypertension and malignant hypertension, and renal damage from these types of hypertension is categorized as benign nephrosclerosis (BN) and malignant nephrosclerosis (MN), respectively. Long-term renal outcomes are much worse in patients with malignant hypertension [16, 17] than in those with benign hypertension [4]. Renal biopsy is useful for the differential diagnosis between HN and primary glomerulonephritis with hypertension [18–20], but only a limited number of studies of biopsy-proven HN have focused on long-term renal outcomes and negative prognostic factors [10–13, 21, 22].

We analyzed data from adult Chinese patients with biopsy-proven HN to investigate the clinico-pathological characteristics and to evaluate the long-term renal survival rates and related risk factors for progression to ESRD. In addition, the clinico-pathological characteristics and prognoses between patients with BN and MN were compared.

## Methods

### Study population

The clinical and renal histopathological data of patients with biopsy-proven HN in Jinling Hospital, Nanjing, from January 2003 to June 2013 were retrospectively reviewed. During the study period, the total number of native kidney biopsies was 31594, from which 411 patients (1.3 %) with a diagnosis of HN were identified. The inclusion criteria were as follows: (1) evidence of hypertension before the detection of proteinuria, hematuria and/or impaired renal function; (2) evidence of HN on renal histology; (3) the lack of any clinical, immunological or histological evidence of other glomerular disease or systemic disorder, such as glomerulonephritis or diabetic nephropathy, and the lack of any defined cause of thrombotic microangiopathy such as hemolytic uremic syndrome or thrombotic thrombocytopenic purpura; (4) an age of between 18 and 65 years; (5) a follow-up period of ≥1.0 year or reaching ESRD within 1 year; and (6) the availability of adequate biopsies (≥8 glomeruli). A total of 194 patients were included in this study.

Indications for renal biopsies included proteinuria (>0.4 g/24 h), hematuria (urine sediment red cell count >100,000 cells/ml) and/or impaired renal function. Renal biopsies were performed after adequate blood pressure control was achieved.

### Clinical and laboratory parameters

The baseline and follow-up data of the patients were obtained by retrospective chart review. The data included gender, age, family history of hypertension, hypertension duration, systolic blood pressure (SBP), diastolic blood pressure (DBP), smoking status, Scr, and uric acid levels, anemia (male: hemoglobin < 120 g/L, female: hemoglobin < 110 g/L), elevated lactate dehydrogenase (LDH) (LDH > 240 U/L), thrombocytopenia (platelets < 100 × 10^9^/L), 24-h urinary protein excretion, microscopic hematuria, hypertensive heart disease, cerebrovascular disease, and retinopathy (using the Keith-Wagener-Barker criteria). The eGFR was calculated using the Chronic Kidney Disease Epidemiology Collaboration (CKD-EPI) equation. Mean arterial pressure (MAP) was defined as the diastolic pressure plus one third of the pulse pressure. For each patient, the highest blood pressure before biopsy and the time-average blood pressure during follow-up, which was defined as the ratio of the area under the curve of MAP during follow-up to the duration of the follow-up [23], were recorded. The number of antihypertensive medications taken during the follow-up period was collected, and oral antihypertensive drugs were categorized into the following classes: angiotensin-converting enzyme inhibitors (ACEIs) and/or angiotensin receptor blockers (ARBs), calcium channel blockers (CCBs), beta blockers (BBs), diuretics and others. Patients were censored at the start of renal replacement therapy or loss to follow-up.

### Renal histopathology

The tissue for light microscopy was serially sectioned, using hematoxylin and eosin, periodic acid-Schiff, methenamine-silver, and Masson trichrome stains. Cryosections were stained with fluorescein isothiocyanate-conjugated rabbit anti-human immunoglobulin G (IgG), IgA, IgM, complement 3 (C3), and C1q. Paraffin sections were stained with fibrinogen. The tissue for electron microscopy was processed according to standard protocols. All biopsy slides were re-reviewed by two pathologists (Dr. Shaoshan Liang and Dr. Dandan Liang) without knowledge of the clinical outcomes. A senior pathologist (Dr. Caihong Zeng) reviewed the slides and made the final decision in cases of disagreement.

HN was divided into two pathological patterns, BN and MN. BN was characterized by arterial or arteriolar hyalinosis, intimal fibrosis, or medial hypertrophy. MN was characterized by fibrinoid necrosis (acute stage) or myointimal cell proliferation, usually with an “onion-skinning” appearance (chronic stage). Samples of concurrent MN and BN lesions were placed into the MN group. The arteries and arterioles were semiquantitatively evaluated for hyalinosis on a scale of 0–2+ (0, absent; 1+, present, nonocclusive of lumen; 2+, present, extensive, and/or impinging on lumen); intimal fibrosis on a scale of 0–4+ (0, no lesions; 1+, minimally recognizable intimal fibrosis; 2+, intimal fibrosis with <25 % luminal occlusion; 3+, intimal fibrosis with 26-50 % luminal occlusion; 4+, advanced lesions with >50 % luminal occlusion); and medial hypertrophy on a scale of 0–2+ (0, absent; 1+, minimal to mild; 2+, moderate to severe). The extents of global glomerulosclerosis, segmental glomerulosclerosis, and glomerular ischemia were expressed as percentages of the total glomeruli. The morphological classification of focal segmental glomerulosclerosis (FSGS) was based on the Columbia classification [24]. The severity of tubular atrophy and interstitial fibrosis (TAIF) was semiquantitatively scored as the percentage of the renal cortical area involvement.

### Outcome

The primary outcome of this study was ESRD, which was defined as eGFR < 15 ml/min/1.73 m^2^, the initiation of chronic renal replacement therapy, or transplantation.

### Statistical analysis

Normally distributed variables were expressed as the mean ± SD and compared using Student’s *t*-test. Non-parametric variables were expressed as medians [interquartile ranges (IQRs)] and compared using the Mann–Whitney-Wilcoxon test. Categorical variables were expressed as the number of positive cases (percentages) and compared using the Pearson *χ*^2^ test. The reproducibility of the pathology variables was evaluated using intraclass correlation coefficients (ICCs). Correlations between the pathology variables were analyzed using the Spearman test. The renal survival rates were estimated using the Kaplan-Meier method, and the log-rank test was used to assess the significance of differences in the Kaplan-Meier survival curves. The Cox proportional hazard model was used to explore the influences of the variables on the occurrence of ESRD. The pathological variables of poor reproducibility (ICC <0.40) were excluded from the model. The variables which were found to be significant (*P* < 0.05) by univariate analysis were included in the multivariate model using backward stepwise method. The assumption of Cox proportional hazards model were assessed using Schoenfeld residuals plots. All P values were two-tailed, and a *P* < 0.05 was considered statistically significant. All analyses were performed using SPSS 18.0 software for Windows (SPSS Inc., Chicago, IL, USA) and *R* software (version 3.2.1).

## Results

### Clinical features

**Fig. 1:**
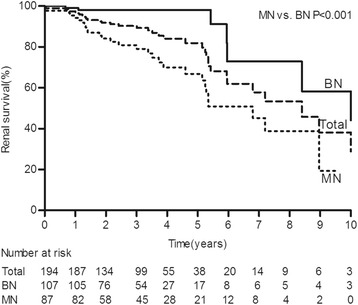
Kaplan-Meier renal survival curves for 194 patients with HN. The 5- and 10-year cumulative renal survival rates after biopsy were 84.5 % and 48.9 %, respectively, for 194 patients with HN. The 5- and 10-year cumulative renal survival rates after biopsy were 98.1 % and 58.3 %, respectively, for the BN group, and 66.8 % and 19.4 %, respectively, for the MN group (log-rank *P* < 0.001). HN, hypertensive nephrosclerosis; BN, benign nephrosclerosis; MN, malignant nephrosclerosis

This study included 194 patients who were predominantly male (82.5 %). The mean age at the time of biopsy was 43.8 ± 4.1 years. The median duration of hypertension was 5.0 years (IQR, 1.0-9.0). The mean SBP and DBP were 195 ± 37 and 126 ± 26 mmHg, respectively. The median Scr level was 1.61 mg/dl (IQR, 1.24-2.27), the median eGFR was 49.6 ml/min/1.73 m^2^ (IQR, 32.8-65.7), and the median proteinuria level was 0.80 g/24 h (IQR, 0.42-1.48). During a median follow-up period of 3.0 years (IQR, 1.8-4.3), 36 patients (18.6 %) developed ESRD (Table 1), and the 5- and 10-year cumulative renal survival rates after renal biopsy, calculated using Kaplan-Meier method, were 84.5 % and 48.9 %, respectively (Fig. 1). The average number of antihypertensive drugs taken during the follow-up period was 2.7 ± 1.3; and 88.7 % of the patients were treated with ACEIs/ARBs, 75.8 % were treated with CCBs, 49.0 % were treated with BBs, and 20.6 % were treated with diuretics.

**Table 1:** Comparisons of the clinical features between the MN and BN groups

	Total patients (*n* = 194)	BN group (*n* = 107)	MN group (*n* = 87)	P
At time of biopsy				
Sex (male:female)	160:34	81:26	79:8	0.006
Age (y)	43.8 ± 4.1	47.4 ± 10.3	39.4 ± 10.7	<0.001
Hypertension family history	114(58.8 %)	59(55.1 %)	55(63.2 %)	0.26
Hypertension duration (y)	5.0(1.0-9.0)	6.0(2.4-10.0)	3.0(0.2-7.0)	<0.001
SBP (mmHg)	195 ± 37	182 ± 32	213 ± 32	<0.001
DBP (mmHg)	126 ± 26	119 ± 23	138 ± 25	<0.001
MAP (mmHg)	150 ± 27	139 ± 24	163 ± 25	<0.001
Current smoker	57(29.4 %)	30(28.0 %)	27(31.0 %)	0.65
Grades of retinopathy				
I-II (%) (n*)	73(50.3 %)(145)	44(58.7 %)(75)	29(41.4 %)(70)	<0.001
III-IV (%) (n*)	42(29.0 %)(145)	8(10.7 %)(75)	34(48.6 %)(70)	
Hypertensive heart disease	110(56.7 %)	42(39.3 %)	68(78.2 %)	<0.001
Hypertensive cerebrovascular disease	18(9.3 %)	12(11.2 %)	6(6.9 %)	0.30
Scr (mg/dl)	1.61(1.24-2.27)	1.35(1.07-1.60)	2.27(1.74-3.14)	<0.001
eGFR (ml/min/1.73 m^2^)	49.6(32.8-65.7)	60.5(48.9-76.5)	34.5(24.2-46.8)	<0.001
eGFR < 60 ml/min/1.73 m^2^	130(67.0 %)	51(47.7 %)	79(90.8 %)	<0.001
Uric acid (μmol/l)	455 ± 109	437 ± 111	482 ± 101	0.004
Anemia	36(18.6 %)	10(9.3 %)	26(29.9 %)	<0.001
Elevated LDH	5(2.6 %)	0(0 %)	5(5.7 %)	-
Thrombocytopenia	1(0.5 %)	0(0 %)	1(1.1 %)	-
Proteinuria (g/24 h)	0.80(0.42-1.48)	0.72(0.38-1.34)	0.89(0.58-1.57)	0.02
Microscopic hematuria	46(23.7 %)	31(29.0 %)	15(17.2 %)	0.06
Follow-up				
No. of antihypertensive drugs	2.7 ± 1.3	2.3 ± 1.1	3.3 ± 1.2	<0.001
Time-average MAP (mmHg) (n*)	129 ± 13(157)	124 ± 11(88)	135 ± 14(69)	<0.001
ESRD	36(18.6 %)	7(6.5 %)	29(33.3 %)	<0.001

Data are presented as the medians (25^th^ and 75^th^ percentiles), the mean ± SD, or the number of positive cases (percentages). n* is the available n for a given value. Where not specified, the available n is the same as the total number of cases in the top row

BN, benign nephrosclerosis; MN, malignant nephrosclerosis; SBP, systolic blood pressure; DBP, diastolic blood pressure; MAP, mean blood pressure; Scr, serum creatinine; eGFR, estimated glomerular filtration rate; LDH, lactate dehydrogenase; ESRD, end-stage renal disease

### Comparisons of clinical features between the MN and BN groups

MN was found in 87 patients (44.8 %), and BN was found in 107 (55.2 %). The comparisons of the clinical features between the BN and MN groups are presented in Table 1. The patients with MN were younger, had a higher male: female ratio, had a shorter duration of hypertension and had higher blood pressure. The MN group had higher incidences of hypertensive heart disease and hypertensive retinopathy than the BN group.

The patients with MN exhibited more severe renal injuries, as indicated by higher median Scr, mean serum uric acid and median proteinuria levels, and a higher incidence of anemia, compared with those with BN.

Although the patients with MN received more antihypertensive medications, the time-average MAP was higher, and more patients progressed to ESRD than those with BN (Table 1). The 5- and 10-year cumulative renal survival rates after biopsy were 98.1 % and 58.3 %, respectively, for the BN group, and 66.8 % and 19.4 %, respectively, for the MN group (log-rank *P* < 0.001) (Fig. 1).

### Comparisons of histological features between the MN and BN groups

The patients with MN exhibited more severe medial hypertrophy, whereas those with BN exhibited a higher hyalinosis score. The intimal fibrosis score was not significantly different between the MN and BN groups. In general, the percentage of segmental glomerulosclerosis was significantly increased in the MN group compared with the BN group. Ninety patients presented with segmental glomerulosclerosis, 54 of which had the perihilar variant. And the MN group had a higher incidence of perihilar variant than the BN group (69.4 % vs. 48.8 %, *P* = 0.047). The percentage of ischemic glomeruli was significantly higher in the MN group than in the BN group, but the percentage of global glomerulosclerosis did not differ between two groups. The percentage of TAIF was significantly greater in the MN group than in the BN group (Table 2).

**Table 2:** Comparisons of the histological features between the MN and BN groups

	Total patients (*n* = 194)	BN group (*n* = 107)	MN group (*n* = 87)	P
Hyalinosis	1.09 ± 0.67	1.25 ± 0.57	0.90 ± 0.73	<0.001
Intimal fibrosis (n*)	1.74 ± 1.18(189)	1.79 ± 1.10(105)	1.68 ± 1.28(84)	0.52
Medial hypertrophy	0.69 ± 0.65	0.42 ± 0.55	1.01 ± 0.62	<0.001
Global glomerulosclerosis (%)	30(18–46)	32(18–45)	28(18–50)	0.63
Segmental glomerulosclerosis (%)	0(0–6)	0(0–5)	3(0–6)	0.04
Ischemic glomeruli (%)	8(0–22)	3(0–9)	20(8–30)	<0.001
TAIF (%)	30(20–60)	30(10–30)	60(40–70)	<0.001

Data are presented as the medians (25^th^ and 75^th^ percentiles), the mean ± SD. n* is the available n for a given value. Where not specified, the available n is the same as the total number of cases in the top row

BN, benign nephrosclerosis; MN, malignant nephrosclerosis; TAIF, tubular atrophy/interstitial fibrosis

### Histological features of MN lesions

Of the 87 patients in the MN group, the incidence of arteriolar involvement was 100 %, whereas that of arterial involvement was 44.0 %. Chronic MN lesions were observed in 94.3 % of the patients in the MN group with arteriolar (81 cases) and arterial (37 cases) involvement. Acute MN lesions were observed in 37.9 % of the patients in the MN group with arteriolar (33 cases) and arterial (1 case) involvement. Twenty-eight patients presented with both chronic and acute MN lesions. Only one case showed glomerular fibrinoid necrosis.

Reproducibility was assessed statistically using ICCs, which are summarized in Table 3.

**Table 3:** ICCs of the HN patients

	ICC
Acute MN lesions	0.66
Chronic MN lesions	0.63
Hyalinosis	0.57
Intimal fibrosis	0.64
Medial hypertrophy	0.40
Global glomerulosclerosis (%)	0.98
Segmental glomerulosclerosis (%)	0.94
Ischemic glomeruli (%)	0.71
TAIF (%)	0.75

Note: an ICC of less than 0.40 is poor reproducibility, 0.40-0.59 is reproducibility, 0.60-0.79 is substantial reproducibility, and 0.80-1 is outstanding reproducibility

MN, malignant nephrosclerosis; TAIF, tubular atrophy/interstitial fibrosis; ICC: intraclass correlation coefficient

Correlations between the pathology variables are shown in Additional file 1: Table S1.

### Clinical and pathological predictors of ESRD

Table 4 shows the MN lesions and renal outcomes for the patients with preserved renal function vs. ESRD. The patients with chronic and acute MN lesions had a higher incidence of progression to ESRD than those with preserved renal function (77.8 % vs. 34.2 %, *P* < 0.001; 33.3 % vs. 13.3 %, *P* = 0.004, respectively). Based on the vessel size, the involvement of both arterioles and arteries in chronic MN lesions and the involvement of arterioles in acute MN lesions were associated with ESRD.

**Table 4:** MN lesions and renal outcomes for patients with preserved renal function vs. ESRD

	Preserved renal function (*n* = 158)	ESRD (*n* = 36)	P
MN	58(36.7 %)	29(80.6 %)	<0.001
Chronic lesions	54(34.2 %)	28(77.8 %)	<0.001
Arteriolar involvement	53(33.5 %)	28(77.8 %)	<0.001
Arterial involvement (n*)	22(14.3 %)(154)	15(42.9 %)(35)	<0.001
Acute lesions	21(13.3 %)	12(33.3 %)	0.004
Arteriolar involvement	21(13.3 %)	12(33.3 %)	0.004
Arterial involvement (n*)	0(0 %)(154)	1(2.9 %)(35)	-

Data are presented as the number of positive cases (percentages). n* is the available n for a given value. Where not specified, the available n is the same as the total number of cases in the top row

MN, malignant nephrosclerosis; ESRD, end-stage renal disease

The univariate Cox regression analysis indicated that the baseline eGFR [hazard ratio (HR), 0.42, 95 % confidence interval (CI), 0.32-0.56 per 10 ml/min/1.73 m^2^ increase, *P* < 0.001], baseline proteinuria (HR, 2.27, 95 % CI, 1.70-3.02 per 1 g/24 h increase, *P* < 0.001), anemia (HR, 4.18, 95 % CI, 2.10-8.33, *P* < 0.001), hyperuricemia (HR, 2.31, 95 % CI, 1.08-4.93, *P* = 0.03), the percentage of global glomerulosclerosis (HR, 1.44, 95 % CI, 1.23-1.70 per 10 % increase, *P* < 0.001), percentage of segmental glomerulosclerosis (HR, 2.02, 95 % CI, 1.31-3.12 per 10 % increase, *P* = 0.001), percentage of TAIF (HR, 1.83, 95 % CI, 1.48-2.25 per 10 % increase, *P* < 0.001), and the presence of MN (HR, 5.51, 95 % CI, 2.27-13.33, *P* < 0.001) were associated with renal outcome (Table 5). These parameters were then considered in the multivariate Cox proportional hazards model, which showed that the baseline eGFR (HR, 0.55, 95 % CI, 0.39-0.77 per 10 ml/min/1.73 m^2^ increase, *P* < 0.001), baseline proteinuria (HR, 1.54, 95 % CI, 1.07-2.21 per 1 g/24 h increase, *P* = 0.02), anemia (HR, 2.28, 95 % CI, 1.04-4.99, *P* = 0.04), percentage of global glomerulosclerosis (HR, 1.33, 95 % CI, 1.12-1.58 per 10 % increase, *P* = 0.001) and percentage of TAIF (HR, 1.48, 95 % CI, 1.08-2.03, *P* = 0.02) were independent predictors of renal outcome (Table 6). Hyperuricemia, the percentage of segmental glomerulosclerosis and the presence of MN were not predictive of renal outcome independently.

**Table 5:** Univariate Cox regression analyses of factors associated with renal survival

	HR (95 % CI)	P
eGFR^a^	0.42(0.32-0.56)	<0.001
Proteinuria^b^	2.27(1.70-3.02)	<0.001
Anemia	4.18(2.10-8.33)	<0.001
Hyperuricemia	2.31(1.08-4.93)	0.03
TAIF^c^	1.83(1.48-2.25)	<0.001
Global glomerulosclerosis^c^	1.44(1.23-1.70)	<0.001
Segmental glomerulosclerosis^c^	2.02(1.31-3.12)	0.001
Presence of MN	5.51(2.27-13.33)	<0.001

^a^per 10 ml/min/1.73 m^2^ increase; ^b^per 1 g/24 h increase; ^c^per 10 % increase

MN, malignant nephrosclerosis; TAIF, tubular atrophy/interstitial fibrosis; HR, hazard ratio; CI, confidence interval

**Table 6:** Multivariate Cox regression analyses of factors associated with renal survival

	HR (95 % CI)	P
eGFR^a^	0.55(0.39-0.77)	<0.001
Proteinuria^b^	1.54(1.07-2.21)	0.02
Anemia	2.28(1.04-4.99)	0.04
TAIF^c^	1.48(1.08-2.03)	0.02
Global glomerulosclerosis^c^	1.33(1.12-1.58)	0.001

^a^per 10 ml/min/1.73 m^2^ increase; ^b^per 1 g/24 h increase; ^c^per 10 % increase

TAIF, tubular atrophy/interstitial fibrosis; HR, hazard ratio; CI, confidence interval

## Discussion

The incidence of hypertension significantly increased from 1991–2009 in China [25], and accordingly, hypertension-induced renal damage also increased. Based on the renal biopsy registry of Jinling Hospital, Nanjing, the incidence of HN increased from 0.45 % in 1979–2002 [26] to 1.3 % in 2003–2013, which was similar to the findings of a recent report from Japan (1.3 %) [27]. Therefore, HN might become a common risk factor for ESRD in China.

Hypertension is categorized as benign hypertension and malignant hypertension, and the renal damage caused by these types of hypertension is classified as BN and MN. MN was observed in 44.8 % of 194 patients with biopsy-proven HN in the present study, which was nearly identical to that reported by Caetano et al. (43 %) [28] and was higher than those reported by other studies (10.3-12.8 %) [20, 29]. The patients in our study were Chinese, whereas those in the other studies were Caucasian or African American. In Caetano’s study [28], the average blood pressure was lower than that in the present study (SBP/DBP 183/117 mmHg vs. 195/126 mmHg), whereas the Scr level was higher (3.28 mg/dl vs. 1.61 mg/dl). In Fogo’s study [20], the average eGFR was higher than that in the present study (51.1 ml/min/1.73 m^2^ vs. 49.6 ml/min/1.73 m^2^). The different incidences of MN in biopsy-proven HN may be associated with variations in ethnicity and patient inclusion criteria.

In the present study, the MN group was younger, displayed higher SBP and DBP levels, had higher median Scr, mean uric acid and median proteinuria levels, and had higher incidences of hypertensive heart disease and retinopathy at baseline than the BN group. In Caetano’s series [28], the MN group was younger, displayed higher DBP and Scr levels, and had a higher incidence of hypertensive retinopathy than the BN group, whereas the uric acid and proteinuria levels were not different between the two groups. In Bohle’s series [29], the MN group was younger and displayed higher SBP and DBP, and Scr and proteinuria levels than the BN group. In Ratschek’s series [30], the MN group displayed higher SBP and DBP and Scr levels, whereas no difference in age was observed. Thus, the previous and present studies show that patients with MN are younger, have a higher Scr level and a higher incidence of hypertensive retinopathy, and have variable uric acid and proteinuria levels compared with those with BN.

In the MN group, 5 patients had elevated LDH and 1 patient had thrombocytopenia at the time of biopsy. In previous retrospective studies of patients with malignant hypertension, 27-44 % of patients have presented with thrombotic microangiopathy [16, 31, 32]. Microangiopathic hemolysis and thrombocytopenia have been reported to resolve within 3–21.7 days and 3–5 days, respectively, in patients with controlled blood pressure [31, 33]. The low incidences of elevated LDH and thrombocytopenia in this study may have been due to the fact that the patients were receiving antihypertensive therapies and had controlled blood pressure on admission to our hospital.

Two pathological patterns based on the vascular lesion, BN and MN, have been described as HN. MN is characterized by fibrinoid necrosis (acute stage) and myointimal cell proliferation, usually with an “onion-skinning” appearance (chronic stage). In the present study, the incidence of chronic-stage MN lesions was as high as 94.3 % in the MN group, whereas that of acute-stage MN lesions was low (37.9 % in the MN group). Furthermore, most patients with acute-stage MN lesions coexisted with chronic-stage MN lesions. These findings are similar to those of previous reports [30, 34]. The acute-stage MN lesions occurred during the more early stage of severe hypertension [34]. All these patients had to achieve adequate blood pressure control before biopsy because of the potential hazard of bleeding complications. The low incidence of acute-stage lesions might have been due to the severe hypertension in the more emergent patients serving as a contraindication of renal biopsy. Segmental fibrinoid necrosis of the glomerular tufts was perceived to be one of the characteristic lesions associated with malignant hypertension [35]. Nevertheless, glomerular fibrinoid necrosis was found in only one case in our study, and it was thus hard to evaluate its relationship with malignant hypertension.

The patients with MN presented with more severe histological lesions than those with BN. In agreement with previous studies [29, 30], the MN group exhibited a higher percentage of ischemic glomeruli (MN vs. BN, 20 % vs. 3 %). MN lesions led to marked narrowing or even occlusion of the lumen and caused the formation of ischemic glomeruli. Furthermore, the percentage of segmental glomerulosclerosis in the MN group was higher (MN vs. BN, 3 % vs. 0 %) and was predominantly of the perihilar type. These data may reflect the fact that the hyperfiltration status of the remaining nephron units was more severe in the MN group than in BN group [36]. The extent of TAIF was significantly higher in the MN group in our study, and previous studies have demonstrated that tubular atrophy and interstitial fibrosis are associated with reduced flow in peritubular capillaries, resulting in hypoxic damage [37, 38]. The MN lesions caused narrowing of the lumen and reduced renal blood flow, leading to hypoxia and ultimately resulting in TAIF. Thus, we speculated that the MN lesions caused the devastating ischemic alterations that resulted in ischemia of glomeruli, segmental glomerulosclerosis, and TAIF. The MN group showed a higher medial hypertrophy score than the BN group in the present study, similar to the results of the study by Caetano [28]. Furthermore, our data showed that hyalinosis was more frequent in the BN group than in the MN group.

HN can lead to ESRD, as shown in Table 7, which summarizes the patient outcomes and prognostic indicators in our study as well as those in previous reports. In the present study, the 5- and 10-year cumulative renal survival rates after biopsy were 98.1 % and 58.3 %, respectively, for the BN group. The 5- and 10-year renal survival rates of BN patients were respectively 80 % and 72 % in Norway [10], 56 % and 35 % in the UK [12], and 35.9 % and 23.6 % in Germany [13]. The patients in our study were younger and had lower baseline Scr levels than those in other studies. In addition, differences in ethnicity and changes in treatment strategies in recent decades might have contributed to the better renal outcomes observed in our study compared with previous studies. With the development of modern antihypertensive drugs, the prognosis of clinical malignant hypertension has improved. We reported the long-term renal outcome of biopsy-proven MN In this study, showing that during a median follow-up period of 3.0 years, 33.3 % of the patients progressed to ESRD in the MN group. Yu et al. reported that a series of MN patients, in which more severe renal involvement was observed, had a worse renal outcome (45.9 % patients progressed to ESRD) during a mean follow-up period of 2.5 years than the present study [39] (Table 7).

**Table 7:** Renal outcomes and prognostic indicators for patients with biopsy-proven HN in the present study and in previous reports

	Case numbers	Sex (male:female)	Age (y)	Scr (eGFR)	Proteinuria (g/24 h)	BP (mmHg)	Duration of follow-up (y)	Renal outcomes	Predictors of renal survival
Takebayashi [21]	590	2.5:1	56.5	1.90(ND)	ND	154(SBP) 87(DBP)	10.1	49.2 % of 345 cases reached endpoint event (Scr ≥3 mg/dl)	Poor or no control of BP, global glomerulosclerosis (>40 %), presence of collapsed glomeruli and/or segmental glomerulosclerosis
Marcantoni [22]	62^a^	1.3:1	58.7	3.40(ND)	1.8	105 ± 3(MAP)	1.9	47.8 % of 23 cases reached ESRD	ND
Wehrmann [13]	170	5.0:1	ND	ND	ND	ND	ND	5- and 10-year renal survival rates were 35.9 % and 23.6 %, respectively	Baseline Scr
Vikse [10]	102	2.0:1	55.4	1.87(ND)	0.4	156 ± 28(SBP); 92 ± 14(DBP)	11.7	5- and 10-year renal survival rates were 80 % and 72 %, respectively	Baseline Scr, proteinuria
Norris [11]	1094^a^	1.6:1	54.6	ND(46.4)	0.3	150 ± 24(SBP); 96 ± 14(DBP)	3.9	13.1 % reached endpoint event (50 % or 25 ml/min per 1.73 m^2^decline in GFR or ESRD)	Baseline proteinuria, GFR, Scr,urea nitrogen, phosphorus
Dasgupta [12]	60	2.0:1	58.0	2.79(36.0)	2.3	179 ± 25(SBP); 105 ± 15(DBP)	6.7	5- and 10-year renal survival rates were 56 % and 35 %, respectively	Baseline Scr, mean SBP and DBP during follow-up (univariate analysis), DBP during follow-up (multivariate analysis)
Yu [39]	61	9.2:1	32.0	6.33(ND)	3.46	ND	2.5	45.9 % reached ESRD	ND
This study	194	4.7:1	43.8	1.61(49.6)	0.80	195 ± 37(SBP); 126 ± 26(DBP)	3.0	5- and 10-year renal survival rates were 84.5 and 48.9 %, respectively	Baseline eGFR, proteinuria, anemia, the percentage of global glomerulosclerosis and TAIF

^a^Most cases underwent renal biopsies

ND, no data; BP, blood pressure; MAP, mean blood pressure; SBP, systolic blood pressure; DBP, diastolic blood pressure; MN, malignant nephrosclerosis; Scr, serum creatinine; eGFR, estimated glomerular filtration rate; ESRD, end-stage renal disease; TAIF, tubular atrophy/interstitial fibrosis

The clinical parameters showed significant prognostic value for patients with HN. Our study confirmed the baseline eGFR and proteinuria level as independent prognostic factors for progression to ESRD [10–13, 40]. In addition, anemia was found to be an independent prognostic factor for renal outcome. The serum uric acid level was higher in the MN group than in the BN group in our study. However, the presence of hyperuricemia, which predicted outcome in univariate Cox analysis, lost its prognostic value in multivariate Cox analysis. In the African American Study of Kidney Disease (AASK) cohort [11], the baseline hematocrit level was significantly associated with the risk for a GFR event or ESRD after adjustments for age, gender, baseline proteinuria, and baseline GFR, whereas no association was observed between serum uric acid level and renal outcome, which was similar to the findings of our study. Further prospective studies are required to confirm whether hyperuricemia is associated with renal outcomes for patients with HN in the Chinese population. Several studies have indicated that an increase in baseline blood pressure [7, 8], treatment-resistant hypertension [6, 9], and increases in SBP and DBP during follow-up [12, 16] are associated with an unfavorable outcome. In the present study, baseline blood pressure was not associated with renal outcome; however, these data are limited by the retrospective nature of this study. Prospective studies are needed to confirm the optimal blood pressure control strategy for patients with HN. Additionally, several studies have indicated that concomitant cardiovascular disease is associated with renal outcome [11, 14, 15]; however, no such associations were observed in the current series of patients.

Some pathological parameters were identified to predict renal outcome. TAIF was one of the prognostic factors identified in the present study that has also been reported as a factor for other renal diseases [41]. Global glomerulosclerosis also exhibited a significant negative prognostic influence in our study. Interestingly, the optimal cutoff of the percentage of global glomerulosclerosis for predicting renal outcome obtained by receiver operating characteristic (ROC) curve analysis was 40 % (data not shown), which was nearly the same as the value obtained from a series of patients with biopsy-proven BN in Japan, in which global glomerulosclerosis (>41 %) at biopsy was found to be an indicator of poor prognosis [21]. These findings suggest that global glomerulosclerosis has a negative prognostic impact on patients with both BN and MN. Correlation analysis of pathology variables demonstrated that there was a strong correlation between the presence of MN and the percentage of TAIF (*r* = 0.643). Thus, the presence of MN predicted the risk for progression to ESRD in univariate Cox analysis, but lost its prognostic value in multivariate Cox analysis.

The limitations of this study must be recognized. The major limitation in this study is its retrospective nature. We cannot rule out the influence of selection bias because some patients, especially those only very mild cases, were unwilling to undergo renal biopsy when HN was diagnosed according to the clinical criteria. In addition, data on patient deaths were incomplete and the candidate factors associated with death were not assessed in this study.

## Conclusions

Our results indicated that patients with MN and BN exhibited significantly different clinico-pathological characteristics. The MN group presented with more severe renal involvement and higher incidences of hypertensive heart disease and retinopathy, received more antihypertensive medications, and had poorer renal outcomes than the BN group. A decreased baseline eGFR, an increased baseline proteinuria level, anemia, increased percentage of global glomerulosclerosis and TAIF were associated with unfavorable renal outcomes in the patients with biopsy-proven HN.

### Ethics approval

The study was approved by the Ethics Committee of Jinling Hospital. No additional administrative permissions were necessary in order to access the clinical and renal histopathological data.

### Data availability statement

All data underlying the findings are within the paper and the supporting information file (Additional file).

## Additional file

Additional file 1: Table S1. Correlations between pathology variables. (DOC 45 kb)

## Abbreviations

ACEIs: angiotensin-converting enzyme inhibitors
ARBs: angiotensin receptor blockers
BBs: beta blockers
BN: benign nephrosclerosis
CCBs: calcium channel blockers
CI: confidence interval
CKD-EPI: chronic kidney disease epidemiology collaboration
DBP: diastolic blood pressure
eGFR: estimated glomerular filtration rate
ESRD: end-stage renal disease
FSGS: focal segmental glomerulosclerosis
HN: hypertensive nephrosclerosis
HR: hazard ratio
ICC: intraclass correlation coefficient
IQR: interquartile range
LDH: lactate dehydrogenase
MAP: mean arterial pressure
MN: malignant nephrosclerosis
ND: no data
ROC: receiver operating characteristic
SBP: systolic blood pressure
Scr: serum creatinine
TAIF: tubular atrophy and interstitial fibrosis
