# Supplementary material for: Clinico-pathological characteristics and outcomes of patients with biopsy-proven hypertensive nephrosclerosis: a retrospective cohort study
Source: BMC Nephrol. 2016 Apr 11;17:42. doi: 10.1186/s12882-016-0254-2 (PMC4827210; doi:10.1186/s12882-016-0254-2)
Supplement: Additional file 1: Table S1. — Correlations between pathology variables. (DOC 45 kb) [file 12882_2016_254_MOESM1_ESM.doc]

Supplemental Table 1. Correlations between pathology variables

|  | Hyalinosis | Intimal  fibrosis | Global  sclerosis | Segmental  sclerosis | Ischemic  glomeruli | TAIF |
| --- | --- | --- | --- | --- | --- | --- |
| MN | -0.256** | - 0.041 | - 0.035 | 0.131 | 0.488** | 0.643** |
| Hyalinosis |  | 0.088 | 0.082 | - 0.021 | - 0.254** | - 0.308** |
| Intimal fibrosis |  |  | - 0.035 | - 0.005 | 0.026 | - 0.003 |
| Global sclerosis |  |  |  | 0.093 | - 0.239** | 0.169* |
| Segmental sclerosis |  |  |  |  | 0.030 | 0.224** |
| Ischemic glomeruli |  |  |  |  |  | 0.532** |

*, Correlation is significant at the 0.05 level (2-tailed); **, Correlation is significant at the 0.01 level (2-tailed).

MN, malignant nephrosclerosis; TAIF, tubular atrophy/interstitial fibrosis.
